# Mental Health and ART Adherence in a Multisite International Study: The Mediating Roles of Social Support and Substance Use in People with HIV

**DOI:** 10.1007/s10461-025-04944-z

**Published:** 2026-01-22

**Authors:** Joseph Awad, Wenxiu Sun, Diane Santa Maria, Rebecca Schnall, Panta Apiruknapanond, Tongyao Wang, Claudia Patricia Valencia, Christine Horvat Davey, Solymar Solis Baez, Emilia Iwu, Motshedisi Sabone, Lufuno Makhado, Yvette P. Cuca, J. Craig Phillips, Inge B. Corless, Sheila Shaibu, Carol Dawson-Rose, Wei-Ti Chen

**Affiliations:** 1 Department of Psychology, University of California Los Angeles, Los Angeles, CA USA; 2 Shanghai Public Health Clinical Center, Fudan University, Shanghai, 201500 CA China; 3 Department of Research, Cizik School of Nursing at UTHealth, 6901 Bertner Avenue, Houston, TX 77030-3901 USA; 4 School of Nursing, Columbia University, 560 West 168, New York, NY 10032 USA; 5 Saint Louis College of Nursing, 19 S Sathon Rd, Yan Nawam, Sathon, Bangkok, 10120 Thailand; 6 School of Nursing, The University of Hong Kong, Academic Building, 3 Sassoon Road, Pokfulam, Hong Kong China; 7 School of Nursing, PROMESA Research Group, Universidad del Valle, Cali, Colombia; 8 Frances Payne Bolton School of Nursing, Case Western Reserve University, 10900 Euclid Ave., Cleveland, OH 44106 USA; 9 Auxilio Mutuo Hospital, PO Box 191227, San Juan, PR 00919 USA; 10 Center for Global Health, School of Nursing, Rutgers University, 180 University Ave, Newark, NJ 07102 USA; 11 Gaborone, Botswana; 12 Department of Public Health, University of Venda, University Road, Private Bag X5050, Thohoyandou, 0950 South Africa; 13 UCSF School of Nursing, 490 Illinois Street, Floor 9, San Francisco, CA 94143 USA; 14 Faculty of Health Sciences, School of Nursing, University of Ottawa, 451 Smyth Road, RGGN 3249A, Ottawa, ON K1S 8M5 Canada; 15 MGH Institute of Health Professions School of Nursing, Boston, USA; 16 School of Nursing and Midwifery, 3, Aga Khan University, Parklands, Nairobi, Kenya; 17 UCSF School of Nursing, 490 Illinois Street, Floor 9, San Francisco, CA 94143 USA; 18 Lulu Wolf Hassenplug Chair in Nursing, Joe C. Wen School of Nursing, University of California Los Angeles, Los Angeles, CA USA; 19 The International Nursing Network for HIV Research, San Francisco, USA

**Keywords:** Mental health, ART adherence, Social support, Substance use, PLHIV

## Abstract

ART adherence, essential to preventing disease progression for people living with HIV (PLHIV), remains challenged by factors like poor mental health condition, insufficient social support, and substance use. This study investigates the roles of social support and substance use as mediators in the relationship between mental health and antiretroviral therapy (ART) adherence among PLHIV in a multisite, international context. Cross-sectional data from *N* = 1598 PLHIV across eight countries were analyzed using parallel mediation analyses in R. Results indicated that both social support and substance use independently mediated the relationship between mental health and ART adherence. Hallucinogens mediated the mental health-adherence link. Cocaine, Amphetamine-type stimulants, inhalants, and opioids directly influenced adherence but did not mediate this relationship. In contrast, no significant effects were observed for tobacco, alcohol, cannabis or sedatives. This large-scale, nurse-led, international study offers critical insights into the roles of social support and substance use as psychosocial influencers in the cascade of care for PLHIV. Clinical implications include prioritizing educative, preventative, and harm-reductive approaches to specific substances that impact ART adherence. Findings may also inform interventions that emphasize strengthening social support to improve adherence in ART and care while addressing substance use as a barrier to care and well-being.

**Supplementary Information:**

The online version contains supplementary material available at https://doi.org/10.1007/s10461-025-04944-z.

## Resumen

La adherencia al tratamiento antirretroviral (TAR), esencial para prevenir la progresión de la enfermedad en personas que viven con VIH (PVV), puede verse afectada por factores como salud mental alterada, apoyo social insuficiente y el consumo de sustancias. Este estudio investiga el comportamiento del apoyo social y el consumo de sustancias como mediadores en la relación entre la salud mental y la adherencia al TAR en unainvestigación internacional multicéntrica. Se analizaron datos de un estudio transversal con1598 PVV en ocho países mediante el análisis de la mediación paralela con el software R. Los resultados indicaron que tanto el apoyo social como el consumo de sustancias median de forma independiente, la relación entre salud mental y adherencia al TAR. Los alucinógenos mediaron el vínculo entre salud mental y adherencia. La cocaína, los estimulantes tipo anfetamina, los inhalantes y los opioides afectaron directamente la adherencia, pero no actuaron como mediadores. No se observaron efectos significativos para el tabaco, el alcohol, el cannabis ni los sedantes. Este estudio internacional, liderado por enfermeras, ofrece información crítica sobre los factores psicosociales que influyen en la cascada de atención para PVV. Las implicaciones clínicas incluyen priorizar enfoques educativos, preventivos y de reducción de daños sobre sustancias específicas que afectan la adherencia al TAR. Los hallazgos también sugieren intervenciones que fortalezcan el apoyo social para mejorar la adherencia al tratamiento y la atención, abordando el consumo de sustancias como una barrera al bienestar y la atención médica.

## Introduction

One of the most important factors that changed the course of the AIDS epidemic was the introduction of antiretroviral therapy (ART) [1]. Recent data reveals that around 39.9 million individuals worldwide were HIV positive in 2023, with 77% of them being on ART [2]. ART medications continue to be recommended for all people living with HIV (PLHIV) [3], and adherence to ART is critical to slow the progression of HIV infection and curb mortality for PLHIV [4]. ART adherence has also been linked to a reduced economic burden of AIDS-defining illnesses [5]. In recent years, differing levels of adherence have been shown to have similar clinical outcomes compared to commonly cited cutoff scores that defined optimal adherence in past eras of HIV treatment [6–8]. However, there remains a need to further examine the antecedents and determinants of the behavioral outcome of adherence itself, given it remains a key modifiable factor to prevent drug resistance and improve the wellbeing of PLHIV [9].

Poor mental health of PLHIV is a major barrier to ART adherence. A substantial amount of evidence has highlighted depression as one mechanism that facilitates the impact of poor mental health on nonadherence [10]. However, little research has focused on social support and substance use as additional mechanisms that buffer and exacerbate nonadherence, respectively. The impact of social support on medication adherence has been well documented in different populations of people with HIV [11–16]. Though most of these studies highlighted mechanisms between social support and adherence, they did not address the role of social support as a mediator itself in relation to mental health. In works where mental health was examined, social support was often posited as the predictor, with mental health acting as the proposed mediator [17, 18], though not all of these models were statistically significant [12]. Given the cross-sectional design of these works, it is important to recognize the atemporality issues that often blur the exact directionality of these relationships, as the field has predominantly been atheoretical or has proposed models requiring further validation [19]. It is plausible that a chronic state of poor mental health in PLHIV may influence their social support, as they may not have the cognitive capacity or social capital to engage in acts that sustain their social relations [11]. Therefore, poor mental health in PLHIV may diminish patients' engagement with their social networks; especially that they still have to navigate the stigma of living with HIV, which may also hinder social relationships.

Substance use has also been documented to have a substantial impact on ART medication adherence [9, 13, 20–23]. PLHIV whose adherence is considered optimal are reported to have a lower proportion of adverse substance use [14]. Particularly, stimulant use (i.e., cocaine or amphetamine) is the biggest influencer of adherence for those who use substances and who have HIV [21, 24, 25]. As recent work has shown, poor mental health may be a confounding variable that affects both non-adherence and substance use [25]. Poor mental health may partially operate through substance use as a coping mechanism for the population, given the substances’ cumulative unique stressors [25]. This would, in turn, impair PLHIV’s ability to optimally adhere to ART. Moreover, while previous studies have reported the impact of specific substances on ART adherence, this work aims to replicate previous findings and further contribute to that literature as well. However, some have found conflicting evidence on the impact of certain substances, especially cannabis [25–31]. This indicates the need to examine the cumulative mediating role of substance use frequency and the specific mediating effects of various substances across cultural and ethnic contexts in the relationship between mental health and adherence.

## Theoretical Framework

The behavioral influences of ART adherence and sustained engagement in care among PLHIV can be broadly elucidated through the Cognitive Behavioral approach [32]. The validity of using this approach as a guiding framework is arguably supported through the successes of cognitive-behavioral interventions and therapies to address adherence among PLHIV [33]. The notion that individuals may be using substances to cope with their mental health can be understood through the lens of The self-medication hypothesis [34] as a behavioral component of emotional regulation [35]. A cognitive-behavioral understanding would posit that a state of poor mental wellbeing likely hinders the dynamic behavioral transactions among individuals by reducing interpersonal functioning and ultimately lessens received social support, which itself has been well established to be important for adherence and behavioral health for PLHIV [36]. Concurrently, using substances that have varying cognitive and emotional influences on PLHIV would also likely impair important behavioral skills and outcomes that drive medication adherence, as also conceptualized by the information-motivation-behavioral skills (IMB) model of adherence [37, 38]. In addition, the IMB model offers a comprehensive framework for understanding and changing health behaviors. It emphasizes that individuals need accurate information, sufficient motivation (personal and social), and the behavioral skills to act effectively. In HIV care, the IMB model helps identify how motivational barriers (e.g., stigma, social norms) and limited coping or communication skills hinder engagement in care. Its structured yet adaptable approach makes it a good fit for designing culturally relevant interventions and understanding behavior change mechanisms. As an extension of the IMB Skills Model, this approach also highlights psychosocial barriers as key antecedents to self-reported ART adherence among PLHIV who use substances [39].

**Fig. 1:**
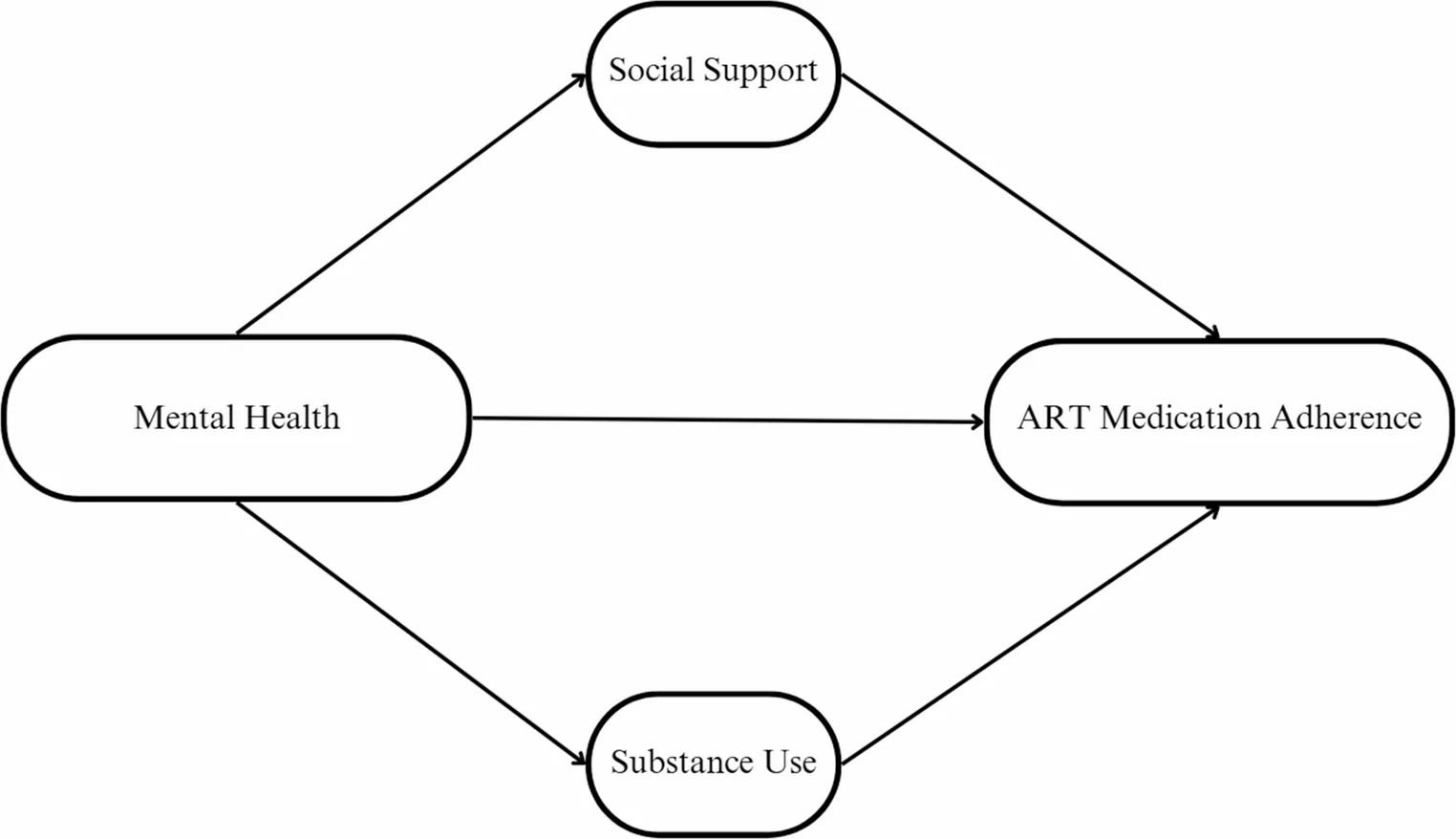
The relationship between mental health and medication adherence as mediated by social support and substance use

In this study, we utilized cross-sectional data from a large-scale, nursing-led, global study with two aims. The primary aim was to assess the impact of mental health on ART adherence as mediated by social support and frequency of substance use among PLHIV, as visualized in Fig. 1. The second aim was to test the individual effect of specific substances for their respective mediating role within that model, together with their individual impact on ART adherence. The substances used in the analyses are tobacco products, alcohol, cannabis, cocaine, amphetamine-type stimulants (speed, diet pills, ecstasy, methamphetamine, cocaine, crack, etc.), inhalants (nitrous, glue, petrol, paint thinner, etc.), sedatives or sleeping pills (Valium, Serepax, Rohypnol, etc.), hallucinogens (LSD, acid, mushrooms, PCP, Special K, etc.), and opioids (heroin, morphine, codeine, etc.). **(Please note that cocaine is listed twice—first after cannabis, and second, contained within amphetamine-type stimulants).

## Methods

### Procedure and Participants

This multisite, international, observational study was designed by the International Nursing Network for HIV Research to achieve the overarching goal of understanding the impact of the COVID-19 pandemic on the lives of PLHIV worldwide [40]. The study included sites in the United States (Boston, Cleveland, Houston, New York, San Francisco) and around the world (Botswana, Canada, China, Colombia, Kenya, Nigeria, Puerto Rico, South Africa, Thailand). In Canada, only (*n* = 3) participants were recruited before the site withdrew from the study, therefore these cases were excluded as they do not sufficiently represent that region. The study employed a convenience sampling method. Participants (N = 1598) were eligible if they self-identified as living with HIV and were at least 21 years old in Puerto Rico or 18 years old in all other locations. Prospective participants were recruited online and in-person and were subsequently screened for meeting the eligibility criteria and for not being a “bot,” by their respective site co-researcher’s team. Online recruitment primarily consisted of efforts via social media and on websites of community organizations that serve PLHIV. In-person recruitment focused on distributing flyers about the study in clinical and community settings. Researchers also directly recruited individuals by approaching them with information about the study at HIV clinics and at support groups’ meetings for PLHIV. Some individuals were informed about this study by their healthcare provider or were referred from other ongoing studies.

Participants provided informed consent in their local language verbally, electronically, or in writing, depending on the site. Individuals who self-administered the study electronically provided consent by clicking “Yes” to the question “Do you consent to participate.” Individuals who completed the study in-person or at a later scheduled time over the telephone met with a study researcher in a private location and physically signed the consent form. At sites that had an approval for a waiver of signed consent, a member of the research team described the study using an information sheet with details about the study, and verbally asked the individual for their consent to participate. Refusal and ineligibility rates were not recorded for this study, and data collection took place from 2021 to 2023. The one-time survey lasted from 15 to 45 min, and all materials were translated to the language of each site. Instruments that were not previously validated in the target language (Chinese, French, Setswana, Sotho, Spanish, Swahili, Tsonga, and Venda) underwent a process of forward translation/back-translation with translators and the study team to establish basic content validity (Brislin, 1970; De Jesús-Espinosa et al., 2024). Participants were able to self-administer the study, in-person or electronically via Qualtrics, or have the survey be interviewer-administered, either in-person or via telephone. Participants were compensated an amount between US$2.26 to US$25.00, as determined by each site. IRB approval was obtained from the University of California, San Francisco [serving as both the Coordinating Center and as a data collection site], and the IRB of each site [Supplement A]. Additional information can be found in the published study protocol [40].

## Materials

### Global Mental Health

Mental Health was assessed using Patient-Reported Outcomes Measurement Information.

System (PROMIS) Global Mental Health scale [41, 42]. This tool was chosen to capture summary information about mental health and general psychological wellbeing, while maintaining minimal respondent burden. Response options ranged from “4-excellent” to “0-poor” for the four items. Sample items include: “In general, how would you rate your mental health, including your mood and your ability to think?” and “In the past 7 days, how often have you been bothered by emotional problems such as feeling anxious, depressed, or irritable?” A final T score was calculated for the analysis, with higher scores reflecting a greater presence of mental health. The scale displays acceptable internal reliability (Cronbach’s *α* = 0.77).

### Social Support

Social support was assessed using the four-item California Health Interview Study instrument [43], which was originally developed by Sherbourne and Stewart [44] as the Medical Outcomes Study Social Support Survey tool. Participants were asked about how frequently someone in their life is available for various things such as to “get together for relaxation?” or “help with daily chores if you are sick?” Response options ranged from “None of the time” to “All of the time” for each of the four items. The scale displays good internal reliability (Cronbach’s α = 0.89).

### Substance Use

Substance use was measured using the ASSIST 10-item measure [45, 46]. Participants were asked to respond to having ever used substances such as cannabis, inhalants, sedatives, among others. If they reported ever having used a given substance, they would subsequently be presented with an item regarding the frequency of use in the past three months. Five response options ranged from “never” to “daily or Almost daily”. The ASSIST measure has been shown to have high internal consistency, and concurrent and discriminant validity for most substances [45–47]. In this sample, however, the items assessing frequency of substance use in the past three months displayed low internal consistency (Cronbach’s *α* = 0.56).

### Medication Adherence

To determine ART adherence, participants were asked to estimate the percentage of the time in the past month in which they took their medication as prescribed. The item was phrased as: “For the past 30 days, what percent of the time were you able to take your HIV medications exactly as your doctor prescribed them?” [48]. Participants were asked to enter a percentage from 0 to 100%. One month recall periods are reported likely to be more accurate than 3- or 7-day periods [49]. Additionally, a continuous measure of adherence as an outcome was chosen to preserve statistical power and better accommodate the diversity of contexts and medication regime types in this global study, as has been done in similar recent work [9].

### Data Analysis

Descriptive analyses were performed using SPSS and parallel mediation analyses were conducted using R [50], while controlling for key sociodemographic covariates that were correlated with the outcome of medication adherence in the bivariate analysis at* p* < 0.10. Bivariate correlations and the process of covariate selection can be found in [Supplement B]. The first model examined the sum score of Global Mental Health as the independent variable, the score of social support as the first mediator, the sum score of all reported substance use frequency in the past three months as the second mediator, in relation to the outcome of ARV medication adherence, while controlling for covariates. Because temporality cannot be fully established in cross-sectional data, we conducted a post-hoc analysis that examined an alternative hypothesis in the proposed mediation of the process by reversing our dependent and independent variables of interest in the main model and comparing model fit statistics with our planned a priori analysis. In following analyses, the score of the frequency of substance use for each individual substance in the past three months was the second mediator, with the same remaining variables. Missing data were handled using Full Information Maximum Likelihood and we assessed statistical significance using bootstrapped confidence intervals that did not cross zero.

## Results

As Table 1 indicates, the sample characteristics display adequate diversity in terms of age (*M* = 44.6, *SD* = 13.7) and education. The majority of participants resided in either the United States (*n* = 770, 31.8%), South Africa (*n* = 285, 17.8%) or China (*n* = 270, 16.9%). Most participants identified as cisgender men (*n* = 904, 56.7%) and cisgender women (*n* = 624, 39.1%). Lastly, 52.7% (*n* = 842) stated not having used substances within the past 3 months, 22.7% (*n* = 363) used one substance, 14.1% (*n* = 225) used two substances, and 10.5% (*n* = 168) used three or more substances within the past 3 months.

**Table 1.** Sample characteristics

		Country															
		Botswana				China				Colombia				Kenya			
		*M*	SD	*n*	%	*M*	SD	*n*	%	*M*	SD	*n*	%	*M*	SD	*n*	%
Global mental health score		46.84	8.39	99		42.76	9.16	265		43.19	8.41	99		39.00	7.17	109	
Social support score		4.16	0.86	100		3.10	1.00	270		3.36	0.97	100		2.98	1.32	110	
Total substance use score		0.22	0.29	95		0.13	0.25	248		0.15	0.30	79		0.04	0.21	86	
Medication adherence %		98	6	100		88	29	235		87	26	95		97	9	105	
95% or higher adherence	Non-adherent			15	15.0%			41	17.4%			36	37.9%			15	14.3%
	Adherent			85	85.0%			194	82.6%			59	62.1%			90	85.7%
	Total			100	100.0%			235	100.0%			95	100.0%			105	100.0%
90% or higher adherence@@	Non-adherent			5	5.0%			34	14.5%			23	24.2%			4	3.8%
	Adherent			95	95.0%			201	85.5%			72	75.8%			101	96.2%
	Total			100	100.0%			235	100.0%			95	100.0%			105	100.0%
80% or higher adherence@@	Non-adherent			2	2.0%			32	13.6%			15	15.8%			3	2.9%
	Adherent			98	98.0%			203	86.4%			80	84.2%			102	97.1%
	Total			100	100.0%			235	100.0%			95	100.0%			105	100.0%
Viral load	Undetectable ( <50 copies/mL)			89	89.0%			259	95.9%			80	95.2%			79	95.2%
	Detectible			11	11.0%			6	2.2%			4	4.8%			4	4.8%
	Don't know			0	0.0%			5	1.9%			0	0.0%			0	0.0%
	Total			100	100.0%			270	100.0%			84	100.0%			83	100.0%
Age	43	10	100		37	12	259		41	15	100		38	10	112		
Residential area	Urban (city)			60	60.0%			248	91.9%			88	88.0%			35	31.3%
	Suburban			20	20.0%			18	6.7%			2	2.0%			73	65.2%
	Rural			20	20.0%			4	1.5%			10	10.0%			4	3.6%
	Total			100	100.0%			270	100.0%			100	100.0%			112	100.0%
Race	African American, Black, African			100	100.0%			0	0.0%			17	17.0%			110	100.0%
	Asian			0	0.0%			270	100.0%			0	0.0%			0	0.0%
	Native Hawaiian/Pacific Islander			0	0.0%			0	0.0%			0	0.0%			0	0.0%
	American Indian or Alaska Native			0	0.0%			0	0.0%			1	1.0%			0	0.0%
	White/Anglo			0	0.0%			0	0.0%			18	18.0%			0	0.0%
	Multiracial			0	0.0%			0	0.0%			52	52.0%			0	0.0%
	Other			0	0.0%			0	0.0%			12	12.0%			0	0.0%
	Total			100	100.0%			270	100.0%			100	100.0%			110	100.0%
Gender	Male			45	45.0%			241	89.6%			46	46.0%			26	23.6%
	Female			54	54.0%			20	7.4%			43	43.0%			83	75.5%
	Transgender Male/Transman/FTM			1	1.0%			2	0.7%			1	1.0%			0	0.0%
	Transgender Female/Transwoman/MTF			0	0.0%			1	0.4%			7	7.0%			0	0.0%
	Genderqueer			0	0.0%			4	1.5%			2	2.0%			1	0.9%
	Other			0	0.0%			1	0.4%			1	1.0%			0	0.0%
	Total			100	100.0%			269	100.0%			100	100.0%			110	100.0%
Education	No schooling			6	6.2%			0	0.0%			0	0.0%			5	4.5%
	Some primary/elementary schooling			12	12.4%			3	1.1%			7	7.0%			16	14.4%
	Completed primary/elementary school			12	12.4%			2	0.7%			5	5.0%			31	27.9%
	Some secondary/high school			32	33.0%			18	6.7%			16	16.0%			20	18.0%
	Completed secondary/high school			18	18.6%			42	15.6%			28	28.0%			19	17.1%
	Some technical, vocational, or university education/training			1	1.0%			17	6.3%			18	18.0%			10	9.0%
	Completed technical or vocational education/training			6	6.2%			43	15.9%			21	21.0%			6	5.4%
	Completed University			6	6.2%			105	38.9%			0	0.0%			3	2.7%
	Completed master’s degree or higher			1	1.0%			40	14.8%			5	5.0%			0	0.0%
	Other			3	3.1%			0	0.0%			0	0.0%			1	0.9%
	Total			97	100.0%			270	100.0%			100	100.0%			111	100.0%
Employment	Working now			28	28.0%			193	71.5%			42	42.0%			48	43.2%
	Temp laid off, sick, maternity			0	0.0%			6	2.2%			1	1.0%			2	1.8%
	Unemployed - looking			7	7.0%			22	8.1%			22	22.0%			47	42.3%
	Retired			0	0.0%			24	8.9%			12	12.0%			1	0.9%
	Disabled			0	0.0%			3	1.1%			1	1.0%			0	0.0%
	Homemaker			6	6.0%			5	1.9%			5	5.0%			4	3.6%
	Student			0	0.0%			15	5.6%			5	5.0%			1	0.9%
	Other			59	59.0%			2	0.7%			12	12.0%			8	7.2%
	Total			100	100.0%			270	100.0%			100	100.0%			111	100.0%

		Country															
		Nigeria				Puerto Rico				South Africa				Thailand			
		*M*	*SD*	*n*	%	*M*	*SD*	*n*	%	*M*	SD	*n*	%	*M*	SD	*n*	%
Global mental health score		46.84	8.39	99		45.59	9.09	99		47.82	11.51	22		46.22	9.99	214	
Social support score		4.16	0.86	100		2.97	1.09	117		3.63	1.17	23		4.57	0.88	285	
Total substance use score		0.22	0.29	95		0.02	0.06	96		0.00	0.00	21		0.15	0.24	275	
**Medication Adherence %**		98	6	100		86	21	107		95	12	23		95	11	271	
**95% or higher adherence**	Non-adherent			15	14.3%			39	36.4%			4	17.4%			80	29.5%
	Adherent			85	85.7%			68	63.6%			19	82.6%			191	70.5%
	Total			100	100.0%			107	100.0%			23	100.0%			271	100.0%
**90% or higher adherence**	Non-adherent			5	3.8%			34	31.8%			2	8.7%			33	12.2%
	Adherent			95	96.2%			73	68.2%			21	91.3%			238	87.8%
	Total			100	100.0%			107	100.0%			23	100.0%			271	100.0%
**80% or higher adherence**	Non-adherent			2	2.9%			28	26.2%			2	8.7%			14	5.2%
	Adherent			98	97.1%			79	73.8%			21	91.3%			257	94.8%
	Total			100	100.0%			107	100.0%			23	100.0%			271	100.0%
**Viral Load**	Undetectable ( <50 copies/mL)			89	95.2%			82	89.1%			20	95.2%			151	99.3%
	Detectible			11	4.8%			10	10.9%			1	4.8%			1	0.7%
	Don't know			0	0.0%			0	0.0%			0	0.0%			0	0.0%
	Total			100	100.0%			92	100.0%			21	100.0%			152	100.0%
**Age**	43	10	100		35	10	119		46	12	23		45	13	285		
**Residential Area**	Urban (city)			60	31.3%			79	68.1%			20	87.0%			2	0.7%
	Suburban			20	65.2%			16	13.8%			2	8.7%			1	0.4%
	Rural			20	3.6%			21	18.1%			1	4.3%			282	98.9%
	Total			100	100.0%			116	100.0%			23	100.0%			285	100.0%
**Race**	African American, Black, African			100	100.0%			61	53.5%			7	30.4%			284	99.6%
	Asian			0	0.0%			2	1.8%			0	0.0%			0	0.0%
	Native Hawaiian/Pacific Islander			0	0.0%			0	0.0%			0	0.0%			0	0.0%
	American Indian or Alaska Native			0	0.0%			0	0.0%			0	0.0%			0	0.0%
	White/Anglo			0	0.0%			0	0.0%			4	17.4%			1	0.4%
	Multiracial			0	0.0%			15	13.2%			12	52.2%			0	0.0%
	Other			0	0.0%			36	31.6%			0	0.0%			0	0.0%
	Total			100	100.0%			114	100.0%			23	100.0%			285	100.0%
**Gender**	Male			45	23.6%			50	42.0%			13	56.5%			115	40.4%
	Female			54	75.5%			59	49.6%			6	26.1%			170	59.6%
	Transgender Male/Transman/FTM			1	0.0%			0	0.0%			0	0.0%			0	0.0%
	Transgender Female/Transwoman/MTF			0	0.0%			1	0.8%			3	13.0%			0	0.0%
	Genderqueer			0	0.9%			1	0.8%			0	0.0%			0	0.0%
	Other			0	0.0%			8	6.7%			1	4.3%			0	0.0%
	Total			100	100.0%			119	100.0%			23	100.0%			285	100.0%
**Education**	No schooling			6	4.5%			5	4.2%			0	0.0%			105	37.0%
	Some primary/elementary schooling			12	14.4%			3	2.5%			0	0.0%			15	5.3%
	Completed primary/elementary school			12	27.9%			4	3.4%			0	0.0%			16	5.6%
	Some secondary/high school			32	18.0%			26	22.0%			2	8.7%			57	20.1%
	Completed secondary/high school			18	17.1%			29	24.6%			7	30.4%			55	19.4%
	Some technical, vocational, or university education/training			1	9.0%			6	5.1%			2	8.7%			19	6.7%
	Completed technical or vocational education/training			6	5.4%			6	5.1%			2	8.7%			5	1.8%
	Completed University			6	2.7%			26	22.0%			5	21.7%			10	3.5%
	Completed master’s degree or higher			1	0.0%			0	0.0%			5	21.7%			2	0.7%
	Other			3	0.9%			13	11.0%			0	0.0%			0	0.0%
	Total			97	100.0%			118	100.0%			23	100.0%			284	100.0%
**Employment**	Working now			28	43.2%			27	23.5%			11	47.8%			103	37.7%
	Temp laid off, sick, maternity			0	1.8%			1	0.9%			2	8.7%			13	4.8%
	Unemployed - looking			7	42.3%			44	38.3%			1	4.3%			96	35.2%
	Retired			0	0.9%			1	0.9%			1	4.3%			23	8.4%
	Disabled			0	0.0%			0	0.0%			4	17.4%			0	0.0%
	Homemaker			6	3.6%			8	7.0%			0	0.0%			27	9.9%
	Student			0	0.9%			20	17.4%			0	0.0%			6	2.2%
	Other			59	7.2%			14	12.2%			4	17.4%			5	1.8%
	Total			100	100.0%			115	100.0%			23	100.0%			273	100.0%

		Country							
		USA				Total			
		*M*	*SD*	*n*	*%*	*M*	*SD*	*N*	*%*
Global mental health score		42.45	10.30	472		43.55	9.60	1481	
Social support score		3.24	1.19	482		3.51	1.21	1590	
Total substance use score		0.39	0.38	412		0.20	0.31	1405	
Medication adherence %		90	20	471		91	20	1511	
95% or higher adherence	Non-adherent			145	30.8%			394	26.1%
	Adherent			326	69.2%			1117	73.9%
	Total			471	100.0%			1511	100.0%
90% or higher adherence	Non-adherent			118	25.1%			263	17.4%
	Adherent			353	74.9%			1248	82.6%
	Total			471	100.0%			1511	100.0%
80% or higher adherence	Non-adherent			70	14.9%			172	11.4%
	Adherent			401	85.1%			1339	88.6%
	Total			471	100.0%			1511	100.0%
Viral load	Undetectable ( <50 copies/mL)			329	81.8%			1175	89.8%
	Detectible			73	18.2%			127	9.7%
	Don't know			0	0.0%			6	0.5%
	Total			402	100.0%			1308	100.0%
Age	52	13	484		44	14	1586		
Residential area	Urban (city)			375	78.5%			939	59.2%
	Suburban			86	18.0%			285	18.0%
	Rural			17	3.6%			363	22.9%
	Total			478	100.0%			1587	100.0%
Race	African American, Black, African			254	52.8%			833	52.5%
	Asian			5	1.0%			381	24.0%
	Native Hawaiian/Pacific Islander			2	0.4%			2	0.1%
	American Indian or Alaska Native			2	0.4%			3	0.2%
	White/Anglo			143	29.7%			166	10.5%
	Multiracial			38	7.9%			117	7.4%
	Other			37	7.7%			85	5.4%
	Total			481	100.0%			1587	100.0%
Gender	Male			322	66.5%			904	56.7%
	Female			144	29.8%			624	39.1%
	Transgender Male/Transman/FTM			3	0.6%			7	0.4%
	Transgender Female/Transwoman/MTF			8	1.7%			20	1.3%
	Genderqueer			5	1.0%			26	1.6%
	Other			2	0.4%			13	0.8%
	Total			484	100.0%			1594	100.0%
Education	No schooling			7	1.4%			128	8.1%
	Some primary/elementary schooling			7	1.4%			71	4.5%
	Completed primary/elementary school			8	1.7%			103	6.5%
	Some secondary/high school			64	13.3%			268	16.9%
	Completed secondary/high school			95	19.7%			295	18.6%
	Some technical, vocational, or university education/training			99	20.5%			181	11.4%
	Completed technical or vocational education/training			41	8.5%			156	9.8%
	Completed University			93	19.3%			249	15.7%
	Completed master’s degree or higher			44	9.1%			97	6.1%
	Other			25	5.2%			42	2.6%
	Total			483	100.0%			1590	100.0%
Employment	Working now			150	31.1%			656	41.6%
	Temp laid off, sick, maternity			13	2.7%			39	2.5%
	Unemployed - looking			75	15.5%			320	20.3%
	Retired			67	13.9%			135	8.6%
	Disabled			129	26.7%			138	8.8%
	Homemaker			15	3.1%			84	5.3%
	Student			6	1.2%			55	3.5%
	Other			28	5.8%			149	9.4%
	Total			483	100.0%			1577	100.0%

**Fig. 2:**
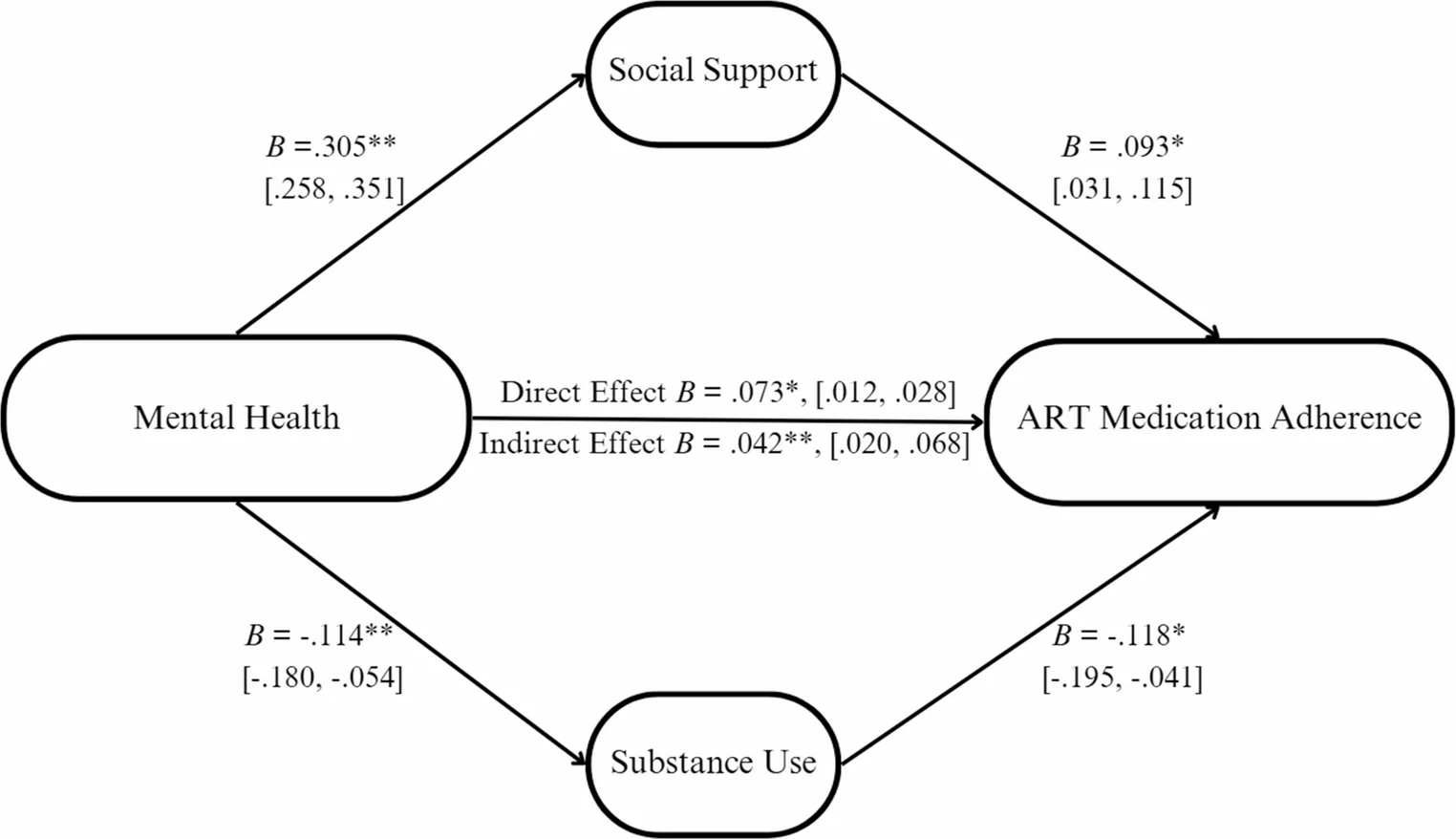
The relationship between mental health and medication adherence as mediated by social support and substance use. *Note* Estimatea and 95% confidence intervals were estimated based on 5000 bootstrap samples. *Denotes *p* < .01 and ** denotes *p* < .001

As seen in Fig. 2 and Table 2, results from a parallel mediation analysis indicated that mental health is indirectly related to ART adherence through its relationship with social support and substance use, respectively and independently. Results of the post-hoc analysis that reversed the dependent and independent variables observed a worse overall model fit (Table 3).

**Table 2.** Parallel mediation analysis of the outcome of adherence, predicted by global mental health (GMH), and mediated by social support (SS) and frequency of total substance use (SU)

Effect type	Variable	BootB	BootSE	p	95% BootCI
Direct (SS)	GMH	.305	.024	.000*	[.258, .351]*
Direct (SU)	GMH	−.114	.032	.000*	[−.180, −.054]*
Direct (Adherence)	SS	.093	.032	.003*	[.031, .155]*
	SU	−.118	.038	.002*	[−.195, −.041]*
	GMH	.073	.030	.013*	[.012, .128]*
Indirect (SS)		.029	.010	.004*	[.010, .048]*
Indirect (SU)		.013	.007	.052	[.003, .030]*
Indirect (Total)		.042	.012	.001*	[.020, .068]*
Total		.115	.029	.000*	[.055, .172]*
*χ*^*2*^(1) = .031*, CFI* = 1.000*, RMSEA* = 0.000*, SRMR* = .000*, AIC* = 10,581.834*, BIC* = 10,876.854*;* *R*^*2*^ = .121*, F(*18, 1164*)* = 8.931*, p* < .000					

All estimates were standardized and based on 5000 bootstrap samples. Analyses controlled for age, country of residence (China, Colombia, Kenya, Nigeria, South Africa, Thailand, and Botswana), residential area (suburban), race (Black/African descent and other), gender (male and other), and employment (retired and student). *Denotes significance at p < .05 or a Confidence Intervals not containing Zero

**Table 3.** Reversed (Post-Hoc) parallel mediation analysis of the outcome of global mental health (GMH), predicted by adherence, and mediated by social support (SS) and frequency of total substance use (SU)

Effect type	Variable	BootB	BootSE	p	95% BootCI
Direct (SS)	Adherence	.123	.027	.000*	[.072, .176]*
Direct (SU)	Adherence	−.134	.044	.002*	[−.229, −.054]*
Direct (GMH)	SS	.376	.031	.000*	[.316, .436]*
	SU	−.111	.030	.000*	[−.170, −.053]*
	Adherence	.073	.029	.013*	[.015, .132]*
Indirect (SS)		.046	.011	.000*	[.027, .068]*
Indirect (SU)		.015	.007	.033*	[.004, .031]*
Indirect (Total)		.061	.014	.000*	[.037, .090]*
Total		.135	.032	.000*	[.072, .199]*
*χ*^*2*^(1) = .834*, CFI* = 1.000*, RMSEA* = 0.000*, SRMR* = .002*, AIC* = 10,843.213*, BIC* = 11,139.163*;* *R*^*2*^ = .178*, F(*18, 1164*)* = 14.02*, p* < .000					

All estimates were standardized and based on 5000 bootstrap samples. Analyses controlled for age, country of residence (China, Colombia, Kenya, Nigeria, South Africa, Thailand, and Botswana), residential area (suburban), race (Black/African descent and other), gender (male and other), and employment (retired and student). *Denotes significance at p < .05 or a Confidence Intervals not containing Zero.

Considering the results of the subsequent individual analyses of the substances, Hallucinogens (Table 4) was the only substance that demonstrated evidence for mediation in the relationship between mental health and adherence. Models with cocaine (*B* = − 0.076, *BootSE* = 0.037, *p* = 0.037, *95% BootCI*: − 0.154 to − 0.012), amphetamines (*B* = − 0.08, *BootSE* = 0.038, *p* = 0.033, *95% BootCI*: − 0.158 to − 0.009), inhalants (*B* = − 0.113, *BootSE* = 0.049, *p* = 0.022, *95% BootCI*: − 0.207 to − 0.015), and opioids (*B* = − 0.057, *BootSE* = 0.033, *p* = 0.090, *95% BootCI*: − 0.134 to − 0.002) did not provide evidence for mediation, yet these substances demonstrated a significant direct impact on adherence in their respective regression models, controlling for the same covariates. Lastly the frequency of use of tobacco (*B* = − 0.04, *BootSE* = 0.026, *p* = 0.128, *95% BootCI*: − 0.095 to 0.009), alcohol (*B* = − 0.043, *BootSE* = 0.028, *p* = 0.134, *95% BootCI*: − 0.101 to 0.012), cannabis (*B* = − 0.056, *BootSE* = 0.033, *p* = 0.08, *95% BootCI*: − 0.123 to 0.006) and sedatives (*B* = − 0.036, *BootSE* = 0.026, *p* = 0.171, *95% BootCI*: − 0.093 to 0.011) did not significantly impact adherence directly in their respective regression models, and there was no evidence to support mediation in the regression models with these substances. All models controlled for age, country of residence (China, Colombia, Kenya, Nigeria, South Africa, Thailand, and Botswana), residential area (suburban), race (Black/African descent and other), gender (male and other), and employment (retired and student).

**Table 4.** Parallel Mediation Analysis of the outcome of Adherence, predicted by Global Mental Health (GMH), and Mediated by Social Support (SS) and Frequency of Hallucinogens Use

Effect type	Variable	*BootB*	*BootSE*	*p*	*95% BootCI*
Direct (SS)	GMH	.305	.024	.000*	[.259, .352]*
Direct (Hallucinogens)	GMH	−.073	.032	.021*	[−.142, −.019]*
Direct (adherence)	SS	.093	.031	.003*	[.032, .154]*
	Hallucinogens	−.092	.038	.014*	[−.181, −.034]*
	GMH	.081	.031	.008*	[.020, .139]*
Indirect (SS)		.028	.010	.003*	[.010, .048]*
Indirect (Hallucinogens)		.007	.005	.157	[.001, .019]*
Indirect (total)		.035	.011	.001*	[.015, .057]*
Total		.116	.029	.000*	[.058, .174]*
*χ*^*2*^(1) = .021*, CFI* = 1.000*, RMSEA* = 0.000*, SRMR* = .000*, AIC* = 11,250.494*, BIC* = 11,545.515*;* *R*^*2*^ = .119*, F(*18, 1286*)* = 9.618*, p* < .000					

All estimates were standardized and based on 5000 bootstrap samples. Analyses controlled for age, country of residence (China, Colombia, Kenya, Nigeria, South Africa, Thailand, and Botswana), residential area (suburban), race (Black/African descent and other), gender (male and other), and employment (retired and student). *Denotes significance at p < .05 or a Confidence Intervals not containing Zero.

## Discussion

Results of this study indicate that the relationship between mental health and ART adherence is mediated by social support and substance use, as hypothesized. Social support was a significant mediator, emphasizing its role in patient engagement in care. Similar findings have been consistently reported in numerous related studies [51, 52]. In addition, this study highlights that PLHIV with stronger support tend to demonstrate better adherence to ART, a finding that aligns with results from other studies [53, 54]. While the mechanisms that facilitate social support’s impact on ART adherence have been established [12], this study offers insight into the impact of mental health on ART adherence through social support in the context of living with HIV. This reduced social connection could, in turn, lead to lower medication adherence due to diminished support, further exacerbating mental health challenges as the disease progresses. Investigators and clinicians should likely prioritize interventions that enhance PLHIV's social networks (i.e., peer-support programs and community engagement initiatives). Notably, the standardized indirect effect of social support was almost two times as large as that of substance use. This may inform a shift in attitudes that focus on the buffering role of social support as a positive key aspect related to enhancing adaptive health behaviors in PLHIV [55].

Poor adherence is associated with worse clinical outcomes, including CD4 count and viral load [56], which may, in turn, influence emotional stresses in PLHIV [57], potentially disrupting social relationships. Thus, poorer social support would play out as established in this analysis, alongside influencing social wellbeing, emotional distress, and depressive symptoms [58]. Combined with our findings of the alternative hypothesis that reverses the variables, findings indicate the need for future longitudinal studies to explore the broader assumption of multidirectional relationships.

Another important takeaway concerning substance use frequency was its mediating role within the model. Similar to social support, it is plausible that people with HIV use substances to cope with mental health issues, as do people without HIV. The underlying mechanism at play may even be depressive symptoms, as their relationship with adherence has been documented [59]. Several studies have shown that opioid dependence and the use of other stimulants increased during the pandemic, while admissions to healthcare facilities declined due to limited access to health institutions [60–62]. Meanwhile, those who used substances more frequently demonstrated poorer ART adherence. This could be attributed to factors such as shelter-in-place orders, hesitation to visit healthcare facilities, simply forgetting to take ART, or being distracted by the substances they were taking.

In the subsequent analyses that homed in on the impact of the use of specific substances, the mediating effect of hallucinogens stands out as a particularly novel insight, given the extremely limited information about its impact on adherence. Other studies corroborate the impact of Amphetamine-type stimulants [25, 31, 63] and opioids [31] on adherence. Integrating harm-reduction strategies like needle exchange programs and medication-assisted therapy into HIV care frameworks may mitigate hallucinogens’ effects. While cocaine and inhalants did not act as mediators in our sample, their direct impact on adherence remains significant and substantial. The relationship between cocaine use and ART adherence has been especially well documented [24, 25, 29, 31]. However, our findings on Cannabis are consistent with previous work [25, 30] but contradict others [26, 28, 29, 31], as some point out its potential symptom-managing benefits [27, 64]. Lastly, other studies on alcohol use have effectively demonstrated its effects on adherence [29, 65, 66], including robust longitudinal approaches [67].

## Strengths and Limitations

There are several limitations in this study. First, the self-report of ART visual analog scale (VAS) adherence is a limitation. Due to variations in ART availability across countries and challenges in ART access during the COVID-19 pandemic, we were unable to design country-specific question for missed doses. For instance, in Botswana, the ART regimen typically consists of a single daily tablet [68], while in China, some participants were prescribed one to multiple pills per day depending on local ART supply [69]. To address these differences, we employed the self-reported ART VAS as a standardized, cross-country measure of adherence, allowing participants to report adherence as a percentage regardless of regimen complexity. Second, given the cross-sectional design of this dataset, we acknowledge the limitations in establishing causal relationships due to the absence of temporal sequencing [70]. Nonetheless, the analysis offers valuable insights into the strength of associations among key variables, which can inform the design of future longitudinal studies. Third, substance use was not explicitly categorized as either recreational or coping related [71]. While self-reported measures of substance use may be subject to certain biases, the use of toxicology screenings was neither practical nor feasible within the scope of this large, international study. Also, not all substances are universally accessible. For instance, amphetamine-type inhalants and opioids are rarely observed in Botswana. Therefore, when interpreting the generalizability of substance use across different study sites, one should be cautious. Fourth, data were not differentiated by language of administration during the data collection. Therefore, we are unable to assess reliability indices to assess the ecological validity of translated measures used in different languages. Fifth, the low Cronbach’s α for the ASSIST scale may be attributed to inconsistencies in translation across languages, which could alter the intended meaning of certain items. Cultural differences may influence how participants interpret and respond to specific questions, contributing to reduced internal consistency. Additionally, the translated instruments used at non-English-speaking sites may have yielded varying Cronbach’s alpha values. Outlier values from these versions could have contributed to lowering the overall Cronbach’s alpha reported for the final English version. However, the team had used a comprehensive framework for cross-cultural translation to best preserve validity that relied on forward/backward translation [72, 73]. Lastly, it is also important to keep in mind the timeline of data collection of this study, as most sites were experiencing “shelter in place” laws in the height of the COVID-19 pandemic, and access to certain substances may have been inhibited. Still, this well-powered study offers comprehensive insights into the dynamic psychosocial and behavioral factors impacting ART adherence.

## Conclusion

This large-scaleinternational study offers critical insights into the roles of social support and substance use as psychosocial influencers in the cascade of care for PLHIV. Clinical implications include practitioners prioritizing educative, preventative, and harm-reduction approaches to specific substances that impact adherence. Findings may also inform interventions that emphasize addressing substance use as a barrier to care while strengthening social support and wellbeing to improve adherence in ART.

## Supplementary Information

Below is the link to the electronic supplementary material.

Supplementary file1 (DOCX 41 KB)
