# Supplementary material for: Mental Health and ART Adherence in a Multisite International Study: The Mediating Roles of Social Support and Substance Use in People with HIV
Source: AIDS Behav. 2026 Jan 22;30(11):3272–87. doi: 10.1007/s10461-025-04944-z (PMC13727863; doi:10.1007/s10461-025-04944-z)
Supplement: Supplementary file 1 [file 10461_2025_4944_MOESM1_ESM.docx]

**Supplement A**

Study Sites and Institutional Review Board / Ethical Approvals

**University of California, San Francisco (UCSF)**

Human Research Protection Program Institutional Review Board

Approval ID: 21-33860

Data collection location: California, USA

**Universidad del Valle Facultad de Salud**

Comité de Ética en Investigación en Salud

Approval ID: 021-021

Data collection location: Colombia

**Case Western Reserve University**

University Hospitals IRB

Approval ID: STUDY20210848

Data collection location: Ohio, USA

**Universidad de Puerto Rico, Recinto de Ciencias Médicas**

Oficina Para la Protección de Participantes Humanos en Investigación, Comité de Derechos Humanos

Approval ID: B3540122

Data collection location: Puerto Rico, USA

**National Health Research Ethics Committee of Nigeria**

Approval ID: NHREC/01/01/2007

Data collection location: Nigeria

**Republic of Botswana Ministry of Health and Wellness**

Health Research and Development Division

Approval ID: HPDME 13/18/1

Data collection location: Botswana

**University of Venda Research and Ethics Committee**

Approval ID: FHS/21/PH/24/2411

Data collection location: South Africa

**University of Ottawa Office of Research Ethics and Integrity**

Approval ID: H-10-21-7383

Data collection location: Canada

**Mass General Brigham IRB**

Approval ID: 2021P002773

Data collection location: Massachusetts, USA

**The Aga Khan University**

Nairobi Institutional Scientific and Ethics Review Committee

Approval ID: 2021/IERC-100

Data collection location: Kenya

**University of California, Los Angeles (UCLA)**

Office of the Human Research Protection Program Institutional Review Board

Approval ID: 21-001233

Data collection location: China

**The University of Texas Health Science Center at Houston**

Committee for the Protection of Human Subjects

Approval ID: HSC-SN-21-0885

Data collection location: Texas, USA

**Columbia University Human Research Protection**

Office Institutional Review Board

Approval ID: IRB-AAAT8779

Data collection location: New York, USA

**Pranangklao Hospital Ethics Committee**

Approval ID: PE6540

Data collection location: Thailand

**Institutional Review Board of the University of Hong Kong**

Hospital Authority Hong Kong West Cluster

Approval ID: UW 22-552

Data collection location: Hong Kong, China

**Supplement B**

Bivariate Analysis between Sociodemographics, Key Predictors, and adherence

I. The following table displays bivariate analysis results. Sociodemographic variables were chosen given homogeneity in data collection across sites, as to not introduce bias, and subsequently dummy-coded.

|  | | Medication Adherence | Social Support | Substance Use | Global Mental Health |
| --- | --- | --- | --- | --- | --- |
| Medication Adherence | Pearson Correlation | 1 |  |  |  |
|  | Sig. (2-tailed) |  |  |  |  |
|  | N | 1511 |  |  |  |
| Social Support | Pearson Correlation | .160 | 1 |  |  |
|  | Sig. (2-tailed) | 0.000 |  |  |  |
|  | N | 1504 | 1590 |  |  |
| Substance Use | Pearson Correlation | -.128 | -.053 | 1 |  |
|  | Sig. (2-tailed) | 0.000 | 0.048 |  |  |
|  | N | 1326 | 1397 | 1405 |  |
| Global Mental Health | Pearson Correlation | .109 | .361 | -.101 | 1 |
|  | Sig. (2-tailed) | 0.000 | 0.000 | 0.000 |  |
|  | N | 1405 | 1475 | 1297 | 1481 |
| Age | Pearson Correlation | .177 | .060 | .097 | 0.020 |
|  | Sig. (2-tailed) | 0.000 | 0.017 | 0.000 | 0.437 |
|  | N | 1500 | 1578 | 1394 | 1469 |
| Country: China | Pearson Correlation | -.066 | -.154 | -.107 | -0.038 |
|  | Sig. (2-tailed) | 0.010 | 0.000 | 0.000 | 0.140 |
|  | N | 1511 | 1590 | 1405 | 1481 |
| Country: Colombia | Pearson Correlation | -.062 | -0.033 | -0.043 | -0.010 |
|  | Sig. (2-tailed) | 0.016 | 0.192 | 0.110 | 0.707 |
|  | N | 1511 | 1590 | 1405 | 1481 |
| Country: Kenya | Pearson Correlation | .077 | -.120 | -.132 | -.133 |
|  | Sig. (2-tailed) | 0.003 | 0.000 | 0.000 | 0.000 |
|  | N | 1511 | 1590 | 1405 | 1481 |
| Country: Nigeria | Pearson Correlation | -.076 | -.128 | -.161 | .057 |
|  | Sig. (2-tailed) | 0.003 | 0.000 | 0.000 | 0.028 |
|  | N | 1511 | 1590 | 1405 | 1481 |
| Country: Puerto Rico | Pearson Correlation | 0.024 | 0.012 | -.080 | .055 |
|  | Sig. (2-tailed) | 0.359 | 0.646 | 0.003 | 0.035 |
|  | N | 1511 | 1590 | 1405 | 1481 |
| Country: South Africa | Pearson Correlation | .077 | .409 | -.089 | .115 |
|  | Sig. (2-tailed) | 0.003 | 0.000 | 0.001 | 0.000 |
|  | N | 1511 | 1590 | 1405 | 1481 |
| Country: Thailand | Pearson Correlation | .059 | 0.020 | -0.044 | 0.017 |
|  | Sig. (2-tailed) | 0.021 | 0.435 | 0.103 | 0.507 |
|  | N | 1511 | 1590 | 1405 | 1481 |
| Country: United States | Pearson Correlation | -.063 | -.147 | .386 | -.078 |
|  | Sig. (2-tailed) | 0.014 | 0.000 | 0.000 | 0.003 |
|  | N | 1511 | 1590 | 1405 | 1481 |
| Country: Botswana | Pearson Correlation | .084 | .140 | 0.011 | .092 |
|  | Sig. (2-tailed) | 0.001 | 0.000 | 0.679 | 0.000 |
|  | N | 1511 | 1590 | 1405 | 1481 |
| Residential area: Urban | Pearson Correlation | -.125 | -.245 | .090 | -.067 |
|  | Sig. (2-tailed) | 0.000 | 0.000 | 0.001 | 0.010 |
|  | N | 1502 | 1580 | 1395 | 1473 |
| Residential area: Suburban | Pearson Correlation | .080 | -.062 | -0.022 | -0.033 |
|  | Sig. (2-tailed) | 0.002 | 0.014 | 0.422 | 0.211 |
|  | N | 1502 | 1580 | 1395 | 1473 |
| Residential area: Rural | Pearson Correlation | .072 | .342 | -.084 | .114 |
|  | Sig. (2-tailed) | 0.005 | 0.000 | 0.002 | 0.000 |
|  | N | 1502 | 1580 | 1395 | 1473 |
| Race: Black / African descent | Pearson Correlation | 0.051 | .218 | 0.044 | .060 |
|  | Sig. (2-tailed) | 0.050 | 0.000 | 0.104 | 0.021 |
|  | N | 1500 | 1579 | 1396 | 1472 |
| Race: Asian | Pearson Correlation | -0.033 | -.128 | -.115 | -0.034 |
|  | Sig. (2-tailed) | 0.199 | 0.000 | 0.000 | 0.197 |
|  | N | 1500 | 1579 | 1396 | 1472 |
| Race: Pacific Islander | Pearson Correlation | -0.030 | -0.030 | .054 | -0.043 |
|  | Sig. (2-tailed) | 0.239 | 0.234 | 0.044 | 0.099 |
|  | N | 1500 | 1579 | 1396 | 1472 |
| Race: Indigenous | Pearson Correlation | -0.022 | -0.022 | 0.004 | -0.042 |
|  | Sig. (2-tailed) | 0.393 | 0.390 | 0.869 | 0.106 |
|  | N | 1500 | 1579 | 1396 | 1472 |
| Race: White | Pearson Correlation | 0.041 | -0.045 | .114 | -0.041 |
|  | Sig. (2-tailed) | 0.116 | 0.076 | 0.000 | 0.114 |
|  | N | 1500 | 1579 | 1396 | 1472 |
| Race: Multiracial | Pearson Correlation | -0.024 | -.110 | 0.009 | -0.006 |
|  | Sig. (2-tailed) | 0.360 | 0.000 | 0.723 | 0.804 |
|  | N | 1500 | 1579 | 1396 | 1472 |
| Race: Other | Pearson Correlation | -.070 | -0.043 | -0.041 | 0.011 |
|  | Sig. (2-tailed) | 0.007 | 0.086 | 0.129 | 0.672 |
|  | N | 1500 | 1579 | 1396 | 1472 |
| Gender: Male | Pearson Correlation | -.083 | -.056 | .086 | 0.023 |
|  | Sig. (2-tailed) | 0.001 | 0.027 | 0.001 | 0.375 |
|  | N | 1508 | 1586 | 1402 | 1477 |
| Gender: Female | Pearson Correlation | .085 | .087 | -.076 | -0.019 |
|  | Sig. (2-tailed) | 0.001 | 0.001 | 0.004 | 0.457 |
|  | N | 1508 | 1586 | 1402 | 1477 |
| Gender: Transgender man | Pearson Correlation | 0.023 | -0.038 | 0.003 | 0.005 |
|  | Sig. (2-tailed) | 0.375 | 0.128 | 0.925 | 0.851 |
|  | N | 1508 | 1586 | 1402 | 1477 |
| Gender: Transgender woman | Pearson Correlation | -0.014 | -0.022 | 0.006 | -0.043 |
|  | Sig. (2-tailed) | 0.581 | 0.384 | 0.826 | 0.100 |
|  | N | 1508 | 1586 | 1402 | 1477 |
| Gender: Genderqueer | Pearson Correlation | 0.039 | -0.036 | -0.024 | 0.004 |
|  | Sig. (2-tailed) | 0.131 | 0.152 | 0.368 | 0.869 |
|  | N | 1508 | 1586 | 1402 | 1477 |
| Gender: Other | Pearson Correlation | -.066 | -.058 | -0.033 | 0.023 |
|  | Sig. (2-tailed) | 0.010 | 0.021 | 0.217 | 0.369 |
|  | N | 1508 | 1586 | 1402 | 1477 |
| Education: None | Pearson Correlation | 0.012 | .191 | -.080 | 0.038 |
|  | Sig. (2-tailed) | 0.641 | 0.000 | 0.003 | 0.147 |
|  | N | 1503 | 1583 | 1397 | 1475 |
| Education: Some primary | Pearson Correlation | 0.038 | 0.002 | -0.019 | -.052 |
|  | Sig. (2-tailed) | 0.143 | 0.928 | 0.485 | 0.044 |
|  | N | 1503 | 1583 | 1397 | 1475 |
| Education: Completed primary | Pearson Correlation | 0.036 | -0.003 | -0.028 | -0.044 |
|  | Sig. (2-tailed) | 0.162 | 0.907 | 0.298 | 0.095 |
|  | N | 1503 | 1583 | 1397 | 1475 |
| Education: Some secondary | Pearson Correlation | -0.005 | 0.006 | -0.006 | 0.003 |
|  | Sig. (2-tailed) | 0.838 | 0.826 | 0.815 | 0.909 |
|  | N | 1503 | 1583 | 1397 | 1475 |
| Education: Completed secondary | Pearson Correlation | -0.040 | 0.021 | 0.001 | 0.048 |
|  | Sig. (2-tailed) | 0.125 | 0.401 | 0.984 | 0.064 |
|  | N | 1503 | 1583 | 1397 | 1475 |
| Education: Some tertiary | Pearson Correlation | -0.008 | -0.037 | .064 | -0.027 |
|  | Sig. (2-tailed) | 0.761 | 0.140 | 0.016 | 0.295 |
|  | N | 1503 | 1583 | 1397 | 1475 |
| Education: Technical | Pearson Correlation | -0.023 | -0.025 | 0.022 | -0.006 |
|  | Sig. (2-tailed) | 0.366 | 0.317 | 0.408 | 0.826 |
|  | N | 1503 | 1583 | 1397 | 1475 |
| Education: University | Pearson Correlation | 0.041 | -.062 | 0.006 | -0.011 |
|  | Sig. (2-tailed) | 0.109 | 0.013 | 0.825 | 0.678 |
|  | N | 1503 | 1583 | 1397 | 1475 |
| Education: Master’s | Pearson Correlation | -0.020 | -0.011 | 0.008 | 0.038 |
|  | Sig. (2-tailed) | 0.434 | 0.654 | 0.770 | 0.145 |
|  | N | 1503 | 1583 | 1397 | 1475 |
| Education: Other | Pearson Correlation | -0.021 | -.112 | 0.032 | -0.017 |
|  | Sig. (2-tailed) | 0.411 | 0.000 | 0.226 | 0.509 |
|  | N | 1503 | 1583 | 1397 | 1475 |
| Employment: Currently employed | Pearson Correlation | -0.006 | 0.022 | -0.042 | .101 |
|  | Sig. (2-tailed) | 0.827 | 0.374 | 0.122 | 0.000 |
|  | N | 1491 | 1569 | 1385 | 1466 |
| Employment: On leave | Pearson Correlation | -0.037 | 0.002 | -0.004 | 0.008 |
|  | Sig. (2-tailed) | 0.159 | 0.940 | 0.869 | 0.749 |
|  | N | 1491 | 1569 | 1385 | 1466 |
| Employment: Unemployed | Pearson Correlation | -0.023 | -0.031 | -0.026 | -.052 |
|  | Sig. (2-tailed) | 0.368 | 0.218 | 0.335 | 0.048 |
|  | N | 1491 | 1569 | 1385 | 1466 |
| Employment: Retired | Pearson Correlation | 0.049 | 0.001 | 0.035 | -0.050 |
|  | Sig. (2-tailed) | 0.060 | 0.964 | 0.194 | 0.057 |
|  | N | 1491 | 1569 | 1385 | 1466 |
| Employment: Disabled | Pearson Correlation | 0.002 | -.078 | .189 | -.055 |
|  | Sig. (2-tailed) | 0.939 | 0.002 | 0.000 | 0.034 |
|  | N | 1491 | 1569 | 1385 | 1466 |
| Employment: Homemaker | Pearson Correlation | 0.030 | .088 | -0.034 | -0.029 |
|  | Sig. (2-tailed) | 0.245 | 0.000 | 0.209 | 0.275 |
|  | N | 1491 | 1569 | 1385 | 1466 |
| Employment: Student | Pearson Correlation | -.088 | -.058 | -.076 | 0.003 |
|  | Sig. (2-tailed) | 0.001 | 0.022 | 0.005 | 0.917 |
|  | N | 1491 | 1569 | 1385 | 1466 |
|  | | | | | |

II. All key variables and sociodemographics that met the threshold of *p <* .10 with the outcome of adherence were entered in a general regression model. If all within the same original categorical variable met the threshold, then one was excluded to act as a reference group. Control variables were removed one by one until all VIF values were <5 starting from the variable with highest VIF value. The following table displays the initial VIF values before any removal.

| Coefficients^a^ | | | | | | | |
| --- | --- | --- | --- | --- | --- | --- | --- |
| Model | Unstandardized Coefficients | | Standardized Coefficients | t | Sig. | Collinearity Statistics | |
|  | B | Std. Error | Beta |  |  | Tolerance | VIF |
| (Constant) | 76.718 | 6.283 |  | 12.210 | 0.000 |  |  |
| Global Mental Health | 0.136 | 0.060 | 0.069 | 2.281 | 0.023 | 0.824 | 1.214 |
| Social Support | 1.724 | 0.528 | 0.107 | 3.266 | 0.001 | 0.707 | 1.414 |
| Substance Use | -7.277 | 1.816 | -0.123 | -4.007 | 0.000 | 0.803 | 1.246 |
| Age | 0.294 | 0.048 | 0.210 | 6.108 | 0.000 | 0.641 | 1.560 |
| Country: China | -0.292 | 4.333 | -0.006 | -0.067 | 0.946 | 0.103 | 9.748 |
| Country: Colombia | -8.607 | 4.593 | -0.110 | -1.874 | 0.061 | 0.219 | 4.575 |
| Country: Kenya | 7.693 | 4.847 | 0.097 | 1.587 | 0.113 | 0.201 | 4.968 |
| Country: Nigeria | -2.422 | 4.880 | -0.029 | -0.496 | 0.620 | 0.221 | 4.517 |
| Country: South Africa | -0.231 | 5.042 | -0.004 | -0.046 | 0.963 | 0.080 | 12.516 |
| Country: Thailand | -2.121 | 4.605 | -0.029 | -0.461 | 0.645 | 0.186 | 5.388 |
| Country: United States | -2.170 | 4.276 | -0.053 | -0.507 | 0.612 | 0.069 | 14.439 |
| Country: Botswana | 5.548 | 4.673 | 0.079 | 1.187 | 0.235 | 0.171 | 5.834 |
| Residential area: Urban | -1.240 | 2.412 | -0.032 | -0.514 | 0.607 | 0.196 | 5.096 |
| Residential area: Suburban | -0.027 | 2.670 | -0.001 | -0.010 | 0.992 | 0.255 | 3.928 |
| Race: Black / African descent | -5.000 | 1.801 | -0.131 | -2.776 | 0.006 | 0.338 | 2.957 |
| Race: Other | -6.764 | 2.801 | -0.080 | -2.415 | 0.016 | 0.692 | 1.445 |
| Gender: Male | -4.974 | 2.945 | -0.129 | -1.689 | 0.092 | 0.129 | 7.735 |
| Gender: Female | -2.586 | 3.010 | -0.066 | -0.859 | 0.390 | 0.128 | 7.810 |
| Gender: Other | -3.494 | 7.526 | -0.014 | -0.464 | 0.643 | 0.822 | 1.217 |
| Employment: Retired | -1.455 | 2.045 | -0.022 | -0.712 | 0.477 | 0.758 | 1.319 |
| Employment: Student | -1.249 | 3.350 | -0.011 | -0.373 | 0.709 | 0.900 | 1.112 |
| a. Dependent Variable: Medication Adherence. | | | | | | | |

III. VIF Values after removing “Country: United States”

| Coefficients^a^ | | | | | | | |
| --- | --- | --- | --- | --- | --- | --- | --- |
| Model | Unstandardized Coefficients | | Standardized Coefficients | t | Sig. | Collinearity Statistics | |
|  | B | Std. Error | Beta |  |  | Tolerance | VIF |
| (Constant) | 74.879 | 5.130 |  | 14.595 | 0.000 |  |  |
| Global Mental Health | 0.137 | 0.060 | 0.070 | 2.299 | 0.022 | 0.825 | 1.212 |
| Social Support | 1.736 | 0.527 | 0.107 | 3.292 | 0.001 | 0.709 | 1.411 |
| Substance Use | -7.430 | 1.790 | -0.126 | -4.150 | 0.000 | 0.825 | 1.212 |
| Age | 0.292 | 0.048 | 0.209 | 6.091 | 0.000 | 0.643 | 1.554 |
| Country: China | 1.644 | 2.052 | 0.033 | 0.801 | 0.423 | 0.457 | 2.187 |
| Country: Colombia | -6.647 | 2.484 | -0.085 | -2.676 | 0.008 | 0.747 | 1.340 |
| Country: Kenya | 9.740 | 2.688 | 0.123 | 3.623 | 0.000 | 0.654 | 1.529 |
| Country: Nigeria | -0.400 | 2.815 | -0.005 | -0.142 | 0.887 | 0.665 | 1.504 |
| Country: South Africa | 1.820 | 3.012 | 0.035 | 0.604 | 0.546 | 0.224 | 4.469 |
| Country: Thailand | -0.182 | 2.568 | -0.003 | -0.071 | 0.944 | 0.596 | 1.677 |
| Country: Botswana | 7.602 | 2.334 | 0.108 | 3.257 | 0.001 | 0.687 | 1.456 |
| Residential area: Urban | -1.229 | 2.411 | -0.032 | -0.510 | 0.610 | 0.196 | 5.096 |
| Residential area: Suburban | -0.037 | 2.669 | -0.001 | -0.014 | 0.989 | 0.255 | 3.927 |
| Race: Black / African descent | -5.110 | 1.788 | -0.134 | -2.859 | 0.004 | 0.343 | 2.914 |
| Race: Other | -6.902 | 2.787 | -0.081 | -2.476 | 0.013 | 0.699 | 1.431 |
| Gender: Male | -5.083 | 2.936 | -0.132 | -1.731 | 0.084 | 0.130 | 7.694 |
| Gender: Female | -2.694 | 3.001 | -0.069 | -0.898 | 0.369 | 0.129 | 7.770 |
| Gender: Other | -3.377 | 7.520 | -0.014 | -0.449 | 0.653 | 0.823 | 1.216 |
| Employment: Retired | -1.462 | 2.044 | -0.023 | -0.715 | 0.474 | 0.758 | 1.319 |
| Employment: Student | -1.286 | 3.348 | -0.011 | -0.384 | 0.701 | 0.900 | 1.111 |
| a. Dependent Variable: Medication Adherence. | | | | | | | |

IV. VIF Values after removing “Gender: Female”

| Coefficients^a^ | | | | | | | |
| --- | --- | --- | --- | --- | --- | --- | --- |
| Model | Unstandardized Coefficients | | Standardized Coefficients | t | Sig. | Collinearity Statistics | |
|  | B | Std. Error | Beta |  |  | Tolerance | VIF |
| (Constant) | 72.690 | 4.513 |  | 16.105 | 0.000 |  |  |
| Global Mental Health | 0.138 | 0.060 | 0.070 | 2.312 | 0.021 | 0.825 | 1.212 |
| Social Support | 1.727 | 0.527 | 0.107 | 3.276 | 0.001 | 0.709 | 1.410 |
| Substance Use | -7.472 | 1.789 | -0.126 | -4.176 | 0.000 | 0.826 | 1.211 |
| Age | 0.289 | 0.048 | 0.206 | 6.041 | 0.000 | 0.647 | 1.545 |
| Country: China | 1.566 | 2.050 | 0.031 | 0.764 | 0.445 | 0.458 | 2.183 |
| Country: Colombia | -6.678 | 2.484 | -0.086 | -2.688 | 0.007 | 0.747 | 1.339 |
| Country: Kenya | 9.576 | 2.682 | 0.121 | 3.571 | 0.000 | 0.657 | 1.522 |
| Country: Nigeria | -0.568 | 2.809 | -0.007 | -0.202 | 0.840 | 0.668 | 1.498 |
| Country: South Africa | 1.654 | 3.006 | 0.032 | 0.550 | 0.582 | 0.225 | 4.452 |
| Country: Thailand | 0.020 | 2.558 | 0.000 | 0.008 | 0.994 | 0.601 | 1.664 |
| Country: Botswana | 7.481 | 2.330 | 0.106 | 3.211 | 0.001 | 0.689 | 1.452 |
| Residential area: Urban | -1.215 | 2.411 | -0.031 | -0.504 | 0.614 | 0.196 | 5.095 |
| Residential area: Suburban | -0.099 | 2.668 | -0.002 | -0.037 | 0.970 | 0.255 | 3.925 |
| Race: Black / African descent | -5.186 | 1.786 | -0.136 | -2.904 | 0.004 | 0.344 | 2.907 |
| Race: Other | -6.923 | 2.787 | -0.082 | -2.484 | 0.013 | 0.699 | 1.431 |
| Gender: Male | -2.669 | 1.178 | -0.069 | -2.266 | 0.024 | 0.808 | 1.238 |
| Gender: Other | -0.971 | 7.026 | -0.004 | -0.138 | 0.890 | 0.942 | 1.061 |
| Employment: Retired | -1.367 | 2.041 | -0.021 | -0.670 | 0.503 | 0.760 | 1.316 |
| Employment: Student | -1.323 | 3.348 | -0.011 | -0.395 | 0.693 | 0.900 | 1.111 |
| a. Dependent Variable: Medication Adherence. | | | | | | | |

V. VIF Values after removing “Residential area: Urban”. All of these covariates were used for control in the mediation and regression models.

| Coefficients^a^ | | | | | | | |
| --- | --- | --- | --- | --- | --- | --- | --- |
| Model | Unstandardized Coefficients | | Standardized Coefficients | t | Sig. | Collinearity Statistics | |
|  | B | Std. Error | Beta |  |  | Tolerance | VIF |
| (Constant) | 71.518 | 3.867 |  | 18.493 | 0.000 |  |  |
| Global Mental Health | 0.139 | 0.059 | 0.070 | 2.329 | 0.020 | 0.826 | 1.211 |
| Social Support | 1.715 | 0.526 | 0.106 | 3.258 | 0.001 | 0.710 | 1.408 |
| Substance Use | -7.453 | 1.789 | -0.126 | -4.167 | 0.000 | 0.826 | 1.210 |
| Age | 0.290 | 0.048 | 0.207 | 6.064 | 0.000 | 0.648 | 1.543 |
| Country: China | 1.517 | 2.047 | 0.030 | 0.741 | 0.459 | 0.459 | 2.178 |
| Country: Colombia | -6.624 | 2.481 | -0.085 | -2.670 | 0.008 | 0.748 | 1.337 |
| Country: Kenya | 9.640 | 2.678 | 0.122 | 3.600 | 0.000 | 0.659 | 1.518 |
| Country: Nigeria | -0.326 | 2.767 | -0.004 | -0.118 | 0.906 | 0.688 | 1.454 |
| Country: South Africa | 2.802 | 1.962 | 0.054 | 1.428 | 0.154 | 0.527 | 1.898 |
| Country: Thailand | 0.062 | 2.556 | 0.001 | 0.024 | 0.981 | 0.602 | 1.662 |
| Country: Botswana | 7.688 | 2.293 | 0.109 | 3.353 | 0.001 | 0.711 | 1.407 |
| Residential area: Suburban | 0.993 | 1.555 | 0.020 | 0.639 | 0.523 | 0.750 | 1.334 |
| Race: Black / African descent | -5.199 | 1.785 | -0.136 | -2.913 | 0.004 | 0.344 | 2.907 |
| Race: Other | -7.050 | 2.774 | -0.083 | -2.541 | 0.011 | 0.704 | 1.420 |
| Gender: Male | -2.670 | 1.178 | -0.069 | -2.267 | 0.024 | 0.808 | 1.238 |
| Gender: Other | -0.631 | 6.992 | -0.003 | -0.090 | 0.928 | 0.951 | 1.052 |
| Employment: Retired | -1.419 | 2.038 | -0.022 | -0.696 | 0.486 | 0.762 | 1.312 |
| Employment: Student | -1.318 | 3.347 | -0.011 | -0.394 | 0.694 | 0.900 | 1.111 |
| a. Dependent Variable: Medication Adherence. | | | | | | | |
